# Reduced astrocytic NF-κB activation by laquinimod protects from cuprizone-induced demyelination

**DOI:** 10.1007/s00401-012-1009-1

**Published:** 2012-07-06

**Authors:** Wolfgang Brück, Ramona Pförtner, Trinh Pham, Jingya Zhang, Liat Hayardeny, Victor Piryatinsky, Uwe-Karsten Hanisch, Tommy Regen, Denise van Rossum, Lars Brakelmann, Karin Hagemeier, Tanja Kuhlmann, Christine Stadelmann, Gareth R. John, Nadine Kramann, Christiane Wegner

**Affiliations:** 1 Department of Neuropathology, University Medical Center, Robert-Koch-Str. 40, 37099 Göttingen, Germany; 2 Department of Neurology, Corinne Goldsmith Dickinson Center for MS, Friedman Brain Institute, Mount Sinai School of Medicine, New York, NY USA; 3 Pharmacology Unit, Global Innovative R&D, Teva Pharmaceutical Industries, Netanya, Israel; 4 Sartorius Stedim Biotech, Göttingen, Germany; 5 Institute of Neuropathology, University Hospital Münster, Münster, Germany

**Keywords:** Demyelination, Laquinimod, Cuprizone, Astrocytes, NF-κB, Multiple sclerosis

## Abstract

Laquinimod (LAQ) is a new oral immunomodulatory compound that reduces relapse rate, brain atrophy and disability progression in multiple sclerosis (MS). LAQ has well-documented effects on inflammation in the periphery, but little is known about its direct activity within the central nervous system (CNS). To elucidate the impact of LAQ on CNS-intrinsic inflammation, we investigated the effects of LAQ on cuprizone-induced demyelination in mice in vivo and on primary CNS cells in vitro. Demyelination, inflammation, axonal damage and glial pathology were evaluated in LAQ-treated wild type and Rag-1-deficient mice after cuprizone challenge. Using primary cells we tested for effects of LAQ on oligodendroglial survival as well as on cytokine secretion and NF-κB activation in astrocytes and microglia. LAQ prevented cuprizone-induced demyelination, microglial activation, axonal transections, reactive gliosis and oligodendroglial apoptoses in wild type and Rag-1-deficient mice. LAQ significantly decreased pro-inflammatory factors in stimulated astrocytes, but not in microglia. Oligodendroglial survival was not affected by LAQ in vitro. Astrocytic, but not microglial, NF-κB activation was markedly reduced by LAQ as evidenced by NF-κB reporter assay. LAQ also significantly decreased astrocytic NF-κB activation in cuprizone-treated mice. Our data indicate that LAQ prevents cuprizone-induced demyelination by attenuating astrocytic NF-κB activation. These effects are CNS-intrinsic and not mediated by peripheral immune cells. Therefore, LAQ downregulation of the astrocytic pro-inflammatory response may be an important mechanism underlying its protective effects on myelin, oligodendrocytes and axons. Modulation of astrocyte activation may be an attractive therapeutic target to prevent tissue damage in MS.

**Electronic supplementary material:**

The online version of this article (doi:10.1007/s00401-012-1009-1) contains supplementary material, which is available to authorized users.

## Introduction

Multiple sclerosis (MS) is the most common chronic neurological disease leading to disability in early to middle adulthood. The pathology of MS is characterized by inflammation-induced demyelination and axonal damage in the central nervous system (CNS). Recent pathological investigations highlight that axonal injury starts early in the disease course [17, 20, 41] and these pathological changes are confirmed by in vivo magnetic resonance imaging (MRI) studies showing early brain volume loss [6, 11, 14]. Acute axonal damage leads to irreversible axonal loss that is thought to be the major correlate of chronic disability in MS [2]. So far, the currently approved treatments for MS mainly target the peripheral immune system. Drugs with myelin- or axon-protecting effects might limit the tissue damage, especially neurodegeneration, and thus prevent accumulation of disability throughout the course of MS. There is a largely unmet need for therapeutics that enter the CNS and directly inhibit myelin and axonal damage.

Laquinimod (LAQ) is a novel immunomodulatory substance that has been shown to be effective, safe and well-tolerated. Phase II studies indicate that LAQ reduces the formation of MRI-active lesions in relapsing-remitting MS [12, 29]. Recent findings from the Phase III study “ALLEGRO” indicate that LAQ has even more pronounced effects on sustained disability progression as well as on brain atrophy compared to its effect on relapses [7, 13]. In this study, LAQ significantly reduced the risk of sustained disability progression and the rate of MRI-measured brain volume loss by about one-third. Moreover, previous studies using whole-body autoradiography demonstrated that 7–8 % of the blood concentration of LAQ penetrates the intact blood–brain barrier and reaches the brain [8]. More evidence of neuroprotective effects of LAQ comes from a recent study indicating that MS patients exhibited higher serum levels of brain-derived neurotrophic factor under LAQ treatment [40]. Combined with the robust clinical effects these data suggest that LAQ might have direct CNS-protective effects in addition to its known peripheral anti-inflammatory properties.

Experimental autoimmune encephalomyelitis (EAE) represents the principle autoimmune animal model of MS and has proven useful in the development of new treatments for this disease [38]. LAQ has been found to inhibit clinical signs of EAE in mice [9] and Lewis rats [47]. In addition, the analysis of cytokine profiles demonstrated that LAQ redirected the cytokine production in favor of the T_H_2/T_H_3 cytokines interleukin 4 (IL-4), interleukin 10 (IL-10) and transforming growth factor-beta (TGF-β) [47]. Recently, LAQ has also been shown to induce type II myeloid cells and regulatory T cells [33]. Compatible with the findings from the ALLEGRO trial, experimental data suggest that LAQ might also exert protective central effects in EAE [46].

Feeding of the copper chelator cuprizone leads to toxic demyelination in the brain of young adult mice. Cuprizone induces oligodendrocyte apoptosis and subsequent demyelination in the near absence of T cells with an intact blood–brain barrier [25]. The exact mechanisms of cuprizone-induced oligodendrocyte death are not well understood. With this model it is possible to study the effect of LAQ on CNS cells without the influence of the peripheral immune system component. Recent findings provide evidence that astrocytic NF-κB activation plays a crucial role for oligodendrocyte damage under cuprizone [31]. Mice deficient in astrocytic NF-κB activation have been reported to show myelin preservation under cuprizone [31].

NF-κB is a transcription factor which is essential for the rapid regulation of cellular responses. In unstimulated cells NF-κB dimers are located in the cytoplasm where they are bound to a family of inhibitor proteins, referred to as IκBs. Activation of NF-κB is initiated by the signal-induced degradation of IκB proteins. This degradation takes place primarily via activation of the IκB kinase (IKK) complex. After degradation of its cytoplasmic inhibitor IκB, the NF-κB proteins translocate into the nucleus and bind to their cognate DNA binding sites to regulate transcription of a large number of genes. This rapid response allows NF-κB to react quickly to stimuli such as bacterial lipopolysaccharides (LPS), tumor necrosis factor alpha (TNFα) and interleukin 1-beta (IL-1β) [28].

To address the action mechanism of LAQ within the CNS, we studied its effects on cuprizone-induced demyelination in mice in vivo and on primary CNS cells in vitro. Here, we report that LAQ is effective in protecting myelin, oligodendrocytes and axons in the cuprizone model. In vitro, LAQ does not affect oligodendroglial survival and displays only minor effects on microglial response. The present study demonstrates that LAQ reduces the inflammatory response in astrocytes by interfering with the astrocytic NF-κB activation in vivo and in vitro. These data point to a new pharmacological target to modulate demyelination-associated pathology. These findings indicate that down-modulation of pro-inflammatory astrocytes by reducing astrocytic NF-κB activation might be an important action mechanism of LAQ.

## Materials and methods

### Test compounds and formulations

LAQ (originally ABR-215062) (RLB#054 M0004) was synthesized at TEVA Pharmaceutical Industries, Ltd. Mice were treated daily with 0, 5 and 25 mg/kg LAQ administered orally from the beginning of the cuprizone feeding. This dose has already been shown to inhibit EAE in C57BL/6 mice [32]. Cuprizone and LAQ treatment was given for 1 or 6 weeks. Control mice received vehicle. In studies in vitro, cell cultures were treated with 50 nM up to 5 μM LAQ or with vehicle as described below. These concentrations are in accordance with the physiological in vivo data in humans [30, 35] and mice [9].

### Mice

8- to 10-week-old male C57BL/6J mice purchased from Charles River (Germany) and female Rag1^−/−^ mice obtained from the animal facility at the University of Göttingen or Jackson Laboratories (USA) were used in these experiments. Animal experiments were conducted in accordance with the European Communities Council Directive of November 24th, 1986 (86/EEC) and were approved by the Government of Lower Saxony, Germany. Each experiment contained at least eight mice per treatment group and was performed at least twice.

### Cuprizone treatment

Cuprizone [oxalic bis(cyclohexylidenehydrazide); Sigma-Aldrich, Germany] is a copper chelator inducing toxic demyelination [3]. Wild type and Rag1^−/−^ mice received a cuprizone diet ad libitum (0.25 %) for 1 week to investigate apoptoses and for 6 weeks to study demyelination of the corpus callosum. Body weights of mice were controlled once weekly.

### Histopathology

Mice were perfused with 4 % paraformaldehyde (PFA). Brains were fixed and embedded in paraffin. Histological evaluation was performed on sections stained with Luxol fast blue-periodic acid-Schiff (LFB-PAS) to determine demyelination. Immunohistochemistry was performed with antibodies against activated microglia (Mac-3, 1:200, clone M3/84, Pharmingen), T cells (CD3, 1:50, clone CD3-12, Serotec), acutely damaged axons (APP, 1:3,000, clone 22C11, Chemicon), glial fibrillary acidic protein (GFAP, 1:200, polyclonal, Dako) and active caspase-3 (active caspase-3, 1:150, clone C92-605, BD Biosciences). Immunofluorescent staining involved antibodies against NF-κB p65 (1:1,000, polyclonal, C-20, Santa Cruz) and GFAP (1:500, clone 134B1, Synaptic Systems). For fluorescence, double-labeling bound antibody was visualized with Streptavidin Cy3-conjugated goat anti-rabbit IgG and Cy2-conjugated goat anti-mouse IgG (both from Jackson ImmunoResearch) with DAPI counterstaining (Sigma-Aldrich).

### Electron microscopy (EM)

In a subset of animals (*n* = 5 per group) EM analysis of the corpus callosum was carried out to confirm the extent of demyelination in mice treated with 0 and 25 mg/kg LAQ. Brains were fixed with 3 % glutaraldehyde in phosphate buffer. Parasagittal slices of 1-mm thickness were obtained. The sections were processed through osmium tetroxide, dehydrated and embedded in Araldite and cut for EM.

### Morphometry and data acquisition

To assess the extent of demyelination, LFB-PAS-stained sections were scored using an extended semi-quantitative scoring system [18]: no (0), minimal (0.5), <33 % (1), 33–66 % (2), and >66 % demyelination (3). GFAP-stained sections were also evaluated semi-quantitatively using the following scoring system: no (0), minimal (1), moderate (2) or severe (3) reactive astrogliosis. The densities of APP-positive axons, Mac3- as well as CD3-positive cells and caspase-3-positive apoptotic cells were counted. Immunofluorescent pictures were taken to assess the proportion of GFAP-positive astrocytes with nuclear NF-κB p65 translocation after 6 weeks of cuprizone. The total number of GFAP-positive astrocytes was determined as well as the number of GFAP-positive astrocytes with nuclear p65 signal to calculate the percentage of GFAP-positive cells with nuclear p65 translocation. All the histological quantifications were carried out blinded.

### Mass spectrometric analysis

Mice were perfused with PBS after 1 or 6 weeks of cuprizone. Brain tissue samples were frozen and stored at −80 °C. Cuprizone was quantified in these brain samples by reverse phase high performance liquid chromatography with MS/MS detection. To this end, 40 μl of 1 μg/ml freshly prepared internal standard solution (omeprazole) and 2 ml of chloroform:acetonitrile (1:1) v/v mixture were added to the frozen mouse brain and homogenized by an ultrasonic homogenizer. The sample was mixed followed by centrifugation. 5 μl of the organic phase was injected onto the HPLC column. The chromatographic separation was performed by gradient elution on an Inertsil ODS-4 chromatographic column (3 μm, 75 × 2.1 mm equipped with guard column Inertsil ODS-4; 3 μm, 10 × 1.5 mm). MS/MS analysis was performed using a TSQ Quantum Ultra AM (Thermo Finnigan) mass spectrometer in positive ionization mode. The following MRM transitions were monitored: *m*/*z* 279.2 → 139.1 for cuprizone and 346.1 → 198.0 for omeprazole.

### Tissue culture and LAQ treatment

Oligodendrocyte precursor cells (OPCs) were prepared by sequential immunopanning and kept under undifferentiating conditions as described earlier [45] until the onset of experiments. OPCs were treated with (up to 10 μM) LAQ for 48 h to assess the effect of LAQ on cellular viability.

Primary cultures of microglial cells were prepared from the brains of newborn C57/BL6 mice as previously described [43]. Cells were plated in 96-well plates for microglial stimulation and NF-κB reporter assays. Microglial cell cultures were pre-incubated with LAQ for 2 h and then treated with either 10 ng/ml LPS, 10 ng/ml TNFα or the combination of 10 ng/ml IL-1β and 10 ng/ml IFNγ for 1 h (NF-κB reporter assay) or 18 h (ELISA) in the absence or presence of LAQ.

Postmortem human tissues were obtained from the Human Tissue Repository at the Albert Einstein College of Medicine and all procedures were approved by the Institutional Clinical Review Committee. Cultures of primary human astrocytes were established and maintained as described [23]. Primary astrocytic cultures from newborn mice were prepared as follows: cerebellum, brainstem and meninges were removed, the remaining brain was trypsinized and cells were taken into culture. After 10 days, microglial cells were shaken off and the remaining astrocytes were trypsinized, centrifuged and transferred into 96-well plates in DMEM containing 10 % FCS, glutamine, penicillin and streptomycin.

### Oligodendroglial viability and apoptosis

To determine cellular viability based on mitochondrial respiration, we performed the MTT [3-(4,5-dimethylthiazol-2-yl)-2,5-diphenyltetrazolium bromide] assay (Sigma), which assesses the ability of metabolically active cells to reduce the tetrazole-dye to purple-colored formazan compounds. To assess direct effects of LAQ on OPCs, cells were incubated with (up to 10 μM) LAQ for 48 h. To determine whether LAQ affects oligodendroglial apoptosis, OPCs were pre-incubated with or without 10 μM LAQ for 6 h, and then (up to 100 nM) staurosporine was added to induced oligodendroglial cell death. Oligodendrocytes were incubated with LAQ for 6 h prior to the addition of staurosporine to the cell cultures. After treatment with staurosporine, cells were washed twice with PBS, and the medium was replaced with medium containing 10 % MTT followed by incubation for 60 min at 37 °C. Cells were lysed using 0.04 N HCl in isopropanol. The absorbance of cell supernatants was measured at 570 nm using a spectrophotometer. Mitochondrial respiration was normalized to that of untreated cells and expressed as percentage of control.

### ELISA and nitrite assay

Murine microglial cells were pre-incubated with 0 and 1 μM LAQ for 2 h and stimulated with 10 ng/ml LPS or the combination of 10 ng/ml IL-1β and IFNγ for 18 h. Culture supernatants were collected and analyzed for the release of cyto- and chemokines by commercial enzyme-linked immunosorbent assay (ELISA) test systems. Levels of IL-6, CCL2, CCL3 and CCL5 were determined using DuoSet ELISA Development Kits (R&D Systems). TNFα levels were measured using an ELISA from BioLegend (San Diego, CA, USA). Absorbance was measured at 450 nm (with a 540 nm reference wavelength) using a microplate reader (Bio-Rad).

Human astrocytic cultures were treated with (0 and up to 5 μM) LAQ and with 10 ng/ml IL-1β for 24 h. Multiplex ELISA was carried out using a commercially available Luminex-based platform (Milliplex) according to the manufacturers’ instructions. After activation with cytokines, levels of nitrite in cell supernatant were measured at 24 h by the Griess reaction as described previously [21].

### Realtime PCR (qPCR)

After treatment of primary human astrocytes with 0 or 1 μM LAQ and cytokines as described above, RNA was harvested using an Absolutely RNA RT-PCR Miniprep Kit (Stratagene, La Jolla, CA, USA). We generated cDNA, and real-time PCR was performed, using a previously published protocol [48]. Each sample transcript was assayed in triplicate and copy numbers were indicated for each transcript.

### Immunoblotting

Primary human astrocytes were incubated with 0 or 100 nM LAQ and stimulated with or without 10 ng/ml IL-1β for 0 and 5 min. SDS-PAGE and western blotting were performed using antibodies for IκBα (Cell Signaling) and β-actin loading control (Invitrogen).

### Imaging flow cytometry analysis of p65 nuclear translocation

After treatment with 0 or 100 nM LAQ, primary human astrocytes were treated with 10 ng/ml IL-1β for 10 min. Cell culture suspensions were fixed and stained for p65 (rabbit, 1:100, Cell Signaling, Beverly), GFAP (mouse, 1:100, DAKO Cytomation) and counterstained for Draq5 (nuclei), and subjected to imaging flow cytometry. Single-cell images were acquired using imaging flow cytometry, and nuclear translocation quantified in approximately 1,000 images captured per sample using IDEAS image analysis software (Amnis).

### Immunofluorescence of cell cultures

Human astrocytes were exposed to 250 nM LAQ for 2 h and treated with 10 ng/ml IL-1β and IFNγ for 24 h. At times specified, cells were fixed and processed for double-immunostaining for GFAP (as above) and β-actin (rabbit, 1:1,000, as above), incubated with the appropriate secondary antibody (1:100, all from Invitrogen) and counterstained with DAPI.

### NF-κB reporter assay

The luciferase reporter assay Cignal Lenti NF-κB Reporter Kit (SABiosciense) was used to monitor the activity of the NF-κB signaling pathway in primary mouse astrocytes and microglia. Cells were transduced with an inducible NF-κB-responsive *firefly* luciferase reporter and a *Renilla reniformis* luciferase normalization reporter using lentiviral vectors. 24 h after transduction with reporter constructs, cultures were pre-incubated with 0 and up to 2.5 μM LAQ for 2 h and subsequently treated with the following cytokines for 1 h: 10 ng/ml TNFα (astrocytes), combination of 10 ng/ml IL-1β and IFNγ (murine astrocytes and microglia) or 10 ng/ml LPS (microglia). Cell lysis and luciferase activity measurement were obtained according to the manufactures’ protocol. Each experiment was measured in triplicates.

### Statistical analysis

Statistical analyses were carried out using the software package SPSS (SPSS 12, Chicago, IL, USA). Histological differences between control mice and treated animals were analyzed by Mann–Whitney *U* tests for nonparametric data and by independent *t* tests for parametric data. For experiments using three or more conditions, data were analyzed using ANOVA plus the Bonferroni post test. Statistical significance was defined as *p* < 0.05.

## Results

### LAQ reduces cuprizone-induced apoptoses

**Fig. 1:**
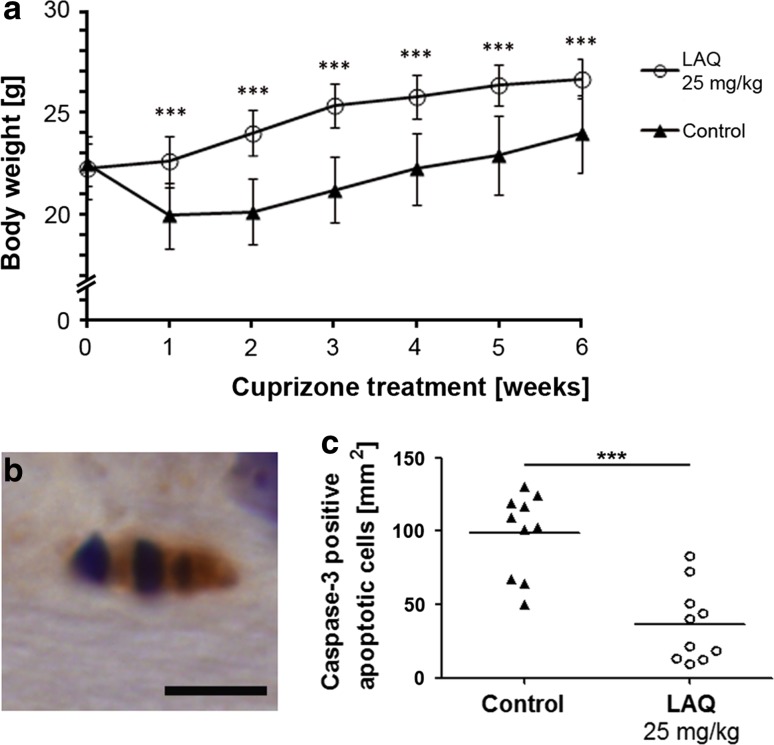
No weight loss and fewer oligodendroglial apoptoses in LAQ-treated mice. **a** At all time points, mice treated with 25 mg/kg LAQ (*n* = 9) display higher body weights than controls (*n* = 10) during 0.25 % cuprizone challenge (*p* < 0.001). Data are expressed as mean values with standard error of the mean (SEM). **b** Apoptotic cells staining positively for active caspase-3 and displaying apoptotic bodies are detected in both groups (*scale bar* 5 μm, shown for an untreated control), but **c** shows that LAQ-treated mice (*n* = 10) display significantly fewer caspase-3-positive apoptoses in the corpus callosum than controls (*n* = 10) after 1 week of 0.25 % cuprizone (*** *p* < 0.001)

To assess the effect of preventive treatment with 25 mg/kg LAQ after cuprizone challenge, controls and LAQ-treated animals were evaluated clinically and histologically. Control mice already showed significantly reduced body weights after 1 week and the following 5 weeks of 0.25 % cuprizone treatment, whereas LAQ-treated mice showed no weight loss (Fig. 1a). To determine whether LAQ exerts effects on oligodendrocytes, we investigated apoptotic cells after 1 week of cuprizone, the time at which oligodendroglial apoptosis is typically observed [24]. Active caspase-3, a marker for cells undergoing apoptosis, was observed in apoptotic cells in both groups (Fig. 1b). The density of caspase-3-positive apoptotic cells in the corpus callosum was significantly lower in LAQ-treated mice than in controls (37 ± 26 vs. 98 ± 28 caspase-3-positive apoptoses per mm^2^; *p* < 0.001) (Fig. 1c).

### LAQ inhibits cuprizone-induced demyelination in a dose-dependent manner

**Fig. 2:**
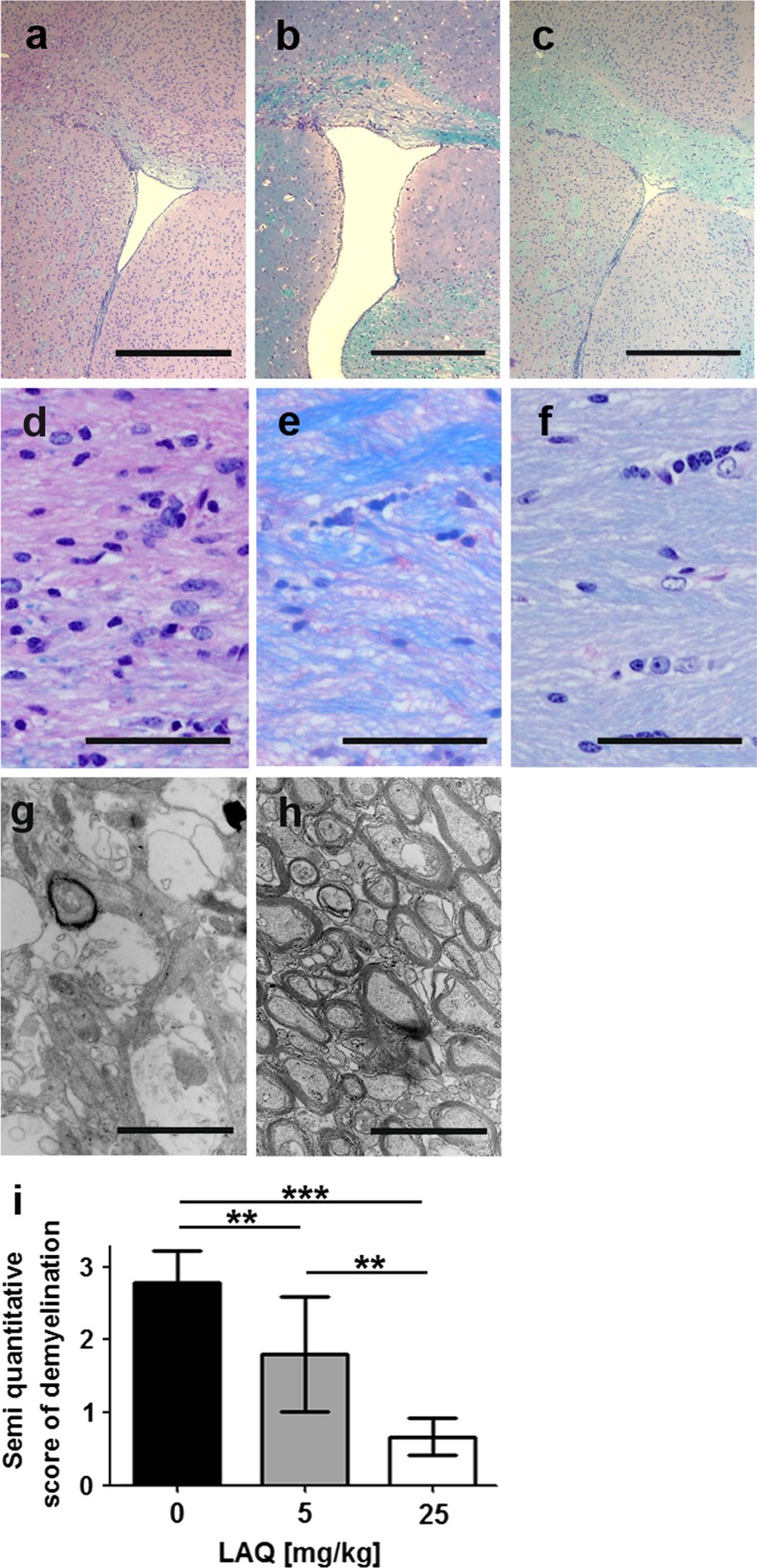
Dose-dependent reduction of callosal demyelination under LAQ after 6 weeks of cuprizone. Demyelination is extensive in controls (*n* = 9) (**a**, **d**, **g**) compared to moderate and minimal demyelination in mice treated with 5 mg/kg (*n* = 10) (**b**, **e**) and 25 mg/kg (*n* = 9) (**c**, **f**, **h**) LAQ, respectively, on LFB-PAS-stained sections (**a**–**f**) and electron microscopic images (**g**, **h**). The semi-quantitative scores for demyelination (**i**) are significantly higher in controls than in both treatment groups. Mice treated with 25 mg/kg LAQ display significantly lower scores than animals treated with 5 mg/kg LAQ (*p < 0.01, ***p < 0.001). *Scale bars* **a**–**c** 500 μm, **d–f** 50 μm, **g**, **h** 2 μm

To test whether preventive treatment with 5 and 25 mg/kg LAQ affects cuprizone-induced demyelination, mice received 0, 5 and 25 mg/kg LAQ during 6 weeks of cuprizone treatment. Demyelination was evaluated in the corpus callosum for each group. Untreated mice displayed extensive callosal demyelination (Fig. 2a, d), whereas mice treated with 25 mg/kg showed mainly intact callosal myelin with only focal signs of demyelination (Fig. 2c, f). Mice receiving the lower dose (5 mg/kg LAQ) showed moderate callosal demyelination (Fig. 2b, e). Electron microscopic evaluation revealed many intact myelin sheaths with little sign of demyelination in mice treated with 25 mg/kg LAQ (Fig. 2h), whereas controls showed numerous demyelinated axons and single remyelinated axons (Fig. 2g). The demyelination scores were significantly higher in untreated mice (score 2.8 ± 0.4) than in mice treated with the lower (score 1.8 ± 0.8, *p* < 0.01) or higher LAQ dose (score 0.6 ± 0.3, *p* < 0.001) (Fig. 2i). Animals treated with 5 mg/kg LAQ displayed significantly higher demyelination scores than mice treated with 25 mg/kg LAQ (*p* < 0.01).

To assess whether the cuprizone levels were comparable in controls and mice treated with 25 mg/kg LAQ, cerebral cuprizone concentrations were measured by mass spectrometric analyses after 1 and 6 weeks. The cerebral cuprizone concentrations did not differ between treated and untreated mice at either time point (supplementary Fig. 1).

### LAQ decreases microglia activation, T cell infiltration, acute axonal damage and reactive astrogliosis in the cuprizone model

**Fig. 3:**
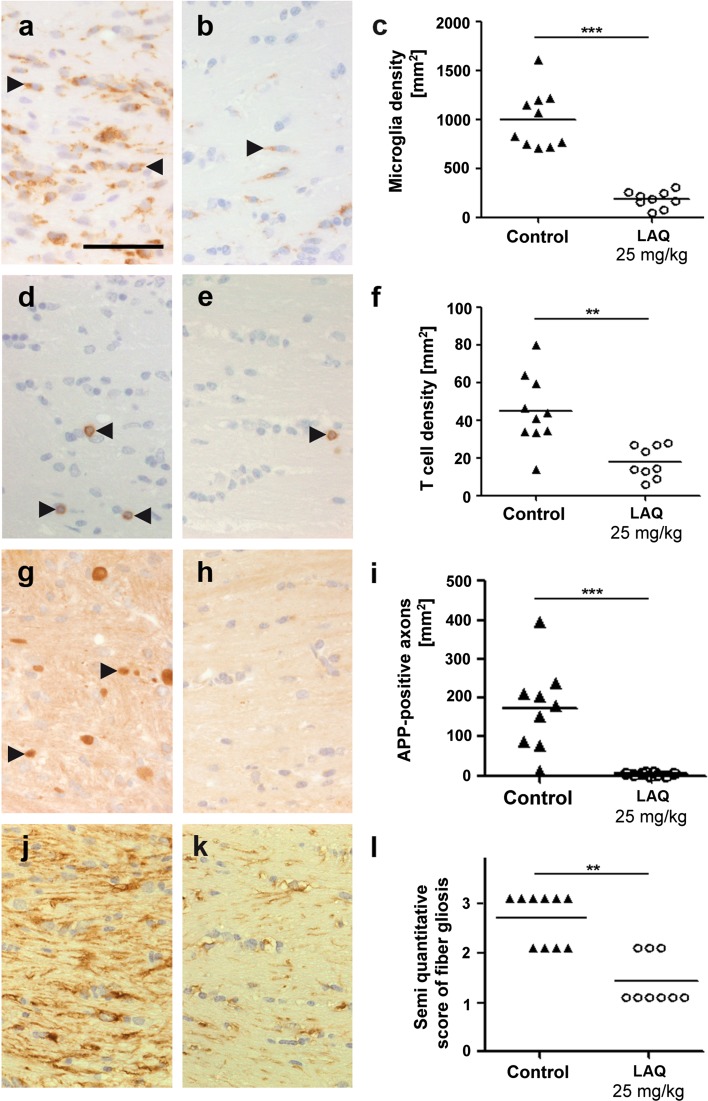
Reduced inflammation, axonal damage and gliosis after LAQ treatment. Compared to untreated controls (*n* = 10) (*left column*), LAQ-treated animals (*n* = 9) (*middle column*) display significantly reduced Mac3-positive microglia (**a**–**c**) and CD3-positive T cell (**d**–**f**) infiltration, APP-positive axonal spheroids (**g**–**i**) and GFAP-positive fibrillary gliosis (**j**–**l**) in the corpus callosum (*scale bars* 50 μm). *Black arrowheads* mark exemplary Mac3-positive microglia (**a**, **b**), CD3-positive T cells (**d**, **e**) and APP-positive axonal spheroids (**g**) (**p < 0.01, ***p < 0.001)

Microglia and T cells were evaluated in untreated controls and mice treated with 25 mg/kg LAQ. The microglia density within the corpus callosum was significantly reduced during LAQ treatment compared to controls (185 ± 84 vs. 1,000 ± 298 mm^2^, *p* < 0.001) (Fig. 3a–c). Staining for CD3 revealed single T cells in the corpus callosum of LAQ-treated mice (Fig. 3e) and a few T cells in control animals (Fig. 3d). LAQ-treated mice displayed significantly fewer T cells than controls (18 ± 9 vs. 45 ± 19 mm^2^, *p* < 0.01) (Fig. 3d–f).

To determine the effect of LAQ on axonal integrity, we analyzed acutely damaged axons characterized by swelling and accumulation of amyloid precursor protein (APP). There was a significant reduction of acute axonal damage under LAQ (Fig. 3h) compared to controls (5 ± 3 vs. 173 ± 111 APP-positive axons per mm^2^; *p* < 0.001) (Fig. 3g, i).

Staining with an antibody against GFAP revealed reduced reactive astrogliosis in LAQ-treated mice (Fig. 3k) compared to prominent fiber gliosis in the corpus callosum of controls (Fig. 3j). The semi-quantitative analysis confirmed this significant difference (Fig. 3l; *p* < 0.01).

### LAQ decreases cuprizone-induced demyelination in Rag1-deficient mice

**Fig. 4:**
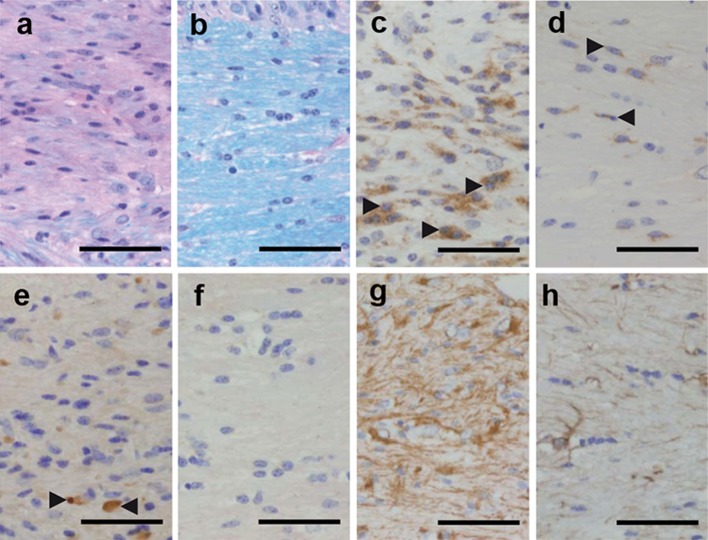
Reduced demyelination, microglia infiltration, acute axonal damage and gliosis in LAQ-treated Rag1^−/−^ mice (*n* = 9) compared to untreated controls (*n* = 10) after 6 weeks of cuprizone. Compared to untreated Rag1^−/−^ mice (**a**, **c**, **e**, **g**) LAQ-treated Rag1^−/−^ mice (**b**, **d**, **f**, **h**) show significantly less demyelination (**a**, **b**), fewer Mac3-positive microglia (**c**, **d**), fewer APP-positive axonal spheroids (**e**, **f**) and less extensive fiber gliosis (**g**, **h**) in the corpus callosum (*scale bars* 50 μm). *Black arrowheads* mark exemplary Mac3-positive microglia (**c**, **d**) and APP-positive axonal spheroids (**e**)

To assess whether the effects of LAQ are independent of T and B cells, we treated Rag1^−/−^ mice, which lack T and B cells, with or without 25 mg/kg LAQ during cuprizone feeding for 6 weeks. Similar to wild type mice, Rag1^−/−^ mice treated with LAQ showed no weight loss compared to the corresponding Rag1^−/−^ control group (supplementary Fig. 2). In comparison to controls, LAQ-treated Rag1^−/−^ mice displayed markedly reduced demyelination in the corpus callosum (Fig. 4a, b), fewer callosal microglia (Fig. 4c, d), fewer APP-positive axonal spheroids (Fig. 4e, f) and less fiber gliosis (Fig. 4g, h).

### LAQ does not affect oligodendroglial survival or modulate microglial response to inflammatory stimuli

**Fig. 5:**
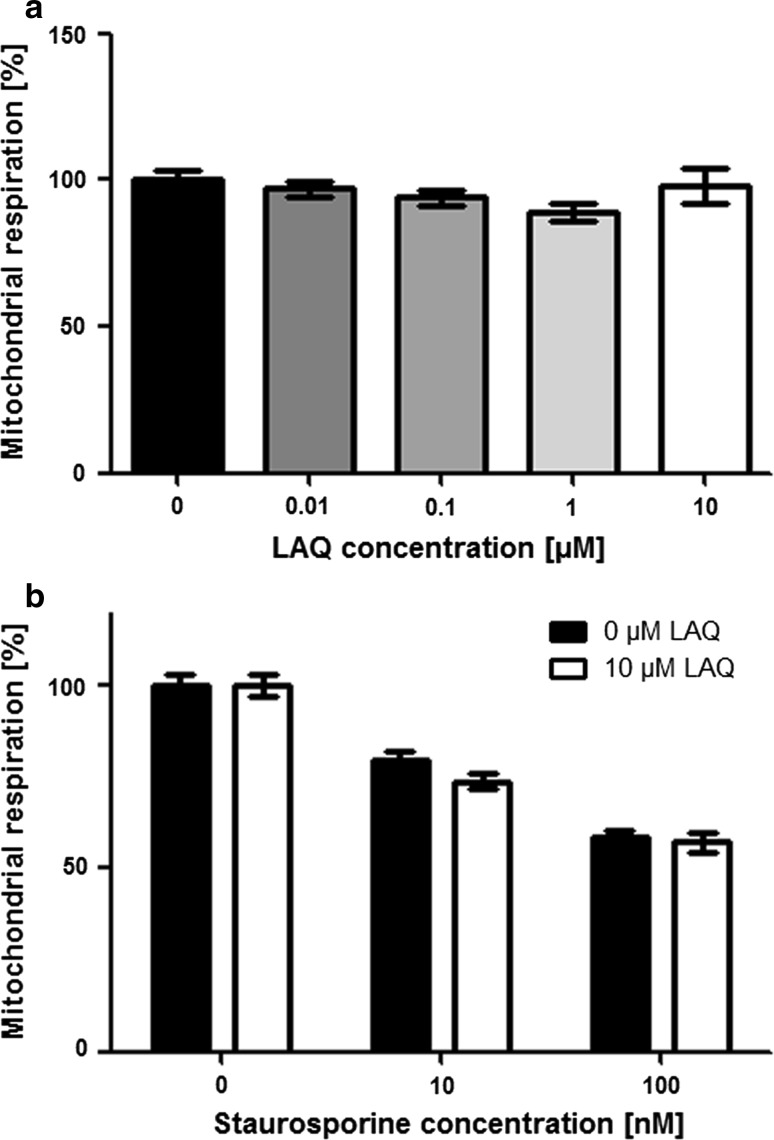
No effect of LAQ on oligodendroglial viability and cell death. **a** OPCs treated with 0, 0.01, 0.1, 1 and 10 μM LAQ for 48 h display similar mitochondrial respiration determined by MTT assays. **b** Treatment with staurosporine for 12 h results in a dose-dependent decrease in mitochondrial respiration indicating cell death. The decrease in mitochondrial respiration was similar in treated (10 μM LAQ) and untreated cells. *Graphs* show mean values with SEM from three independent experiments

We performed in vitro experiments in oligodendroglial precursor cells (OPCs) to assess whether LAQ influences oligodendroglial survival and apoptosis. Treatment with (up to 10 μM) LAQ for 48 h did not affect cell viability measured by MTT assays (Fig. 5a). To determine if LAQ affects oligodendroglial cell death, OPCs were treated with 10 μM LAQ and incubated with increasing concentrations of staurosporine. Oligodendroglial cell death was induced in a dose-dependent manner by staurosporine (Fig. 5b). Staurosporine-induced cell death did not differ between LAQ-treated and untreated OPCs (Fig. 5b).

**Fig. 6:**
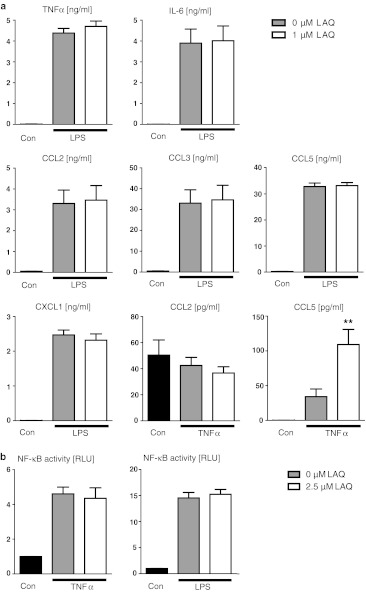
No marked effect of LAQ on microglial cytokine secretion (**a**) and NF-κB activation (**b**). **a** Microglial cultures were exposed to LPS (10 ng/ml) or TNFα (10 ng/ml) for 18 h in the absence or presence of 1 μM LAQ. LAQ-treated cells also received a pre-incubation with LAQ for 2 h. Cyto- and chemokines were determined in the supernatant. LPS and TNFα stimulation significantly increased the cytokine levels compared to unstimulated controls (*p* < 0.05 for TNFα-induced CCL5, *p* < 0.001 for remaining cytokines shown). Cells with and without LAQ (pre-) treatment showed similar levels of LPS-induced TNFα, IL-6, CXCL1, CCL2, CCL3 and CCL5. After TNFα stimulation, LAQ-treated cells showed higher CCL5 levels (*p < 0.01), but similar CCL2 release. *Graphs* show mean values with SEM from two independent experiments. **b** Treatment with 2.5 μM LAQ has no effect on NF-κB activity in stimulated primary mouse microglia assessed by a NF-κB reporter assay. Cells stimulated with LPS or TNFα were pre-treated with and without LAQ. NF-κB activity was measured luminometrically (*RLU* relative light units). Cells exposed to these cytokines displayed a significant NF-κB activation that was not reduced by the (pre-) treatment with LAQ. *Graphs* show mean values with SEM from three experiments

To determine whether LAQ affects microglial responses to inflammatory challenges, primary cultures were pre-treated with 0 and 1 μM LAQ and then stimulated with LPS (as a representative TLR agonist) or TNFα (as a cytokine with key roles in inflammation) in the absence or presence of LAQ (1 μM). LAQ treatment alone did not affect cytokine production. LPS stimulation significantly increased TNFα, IL-6, CXCL1, CCL2, CCL3 and CCL5 release compared to unstimulated controls (*p* < 0.001) (Fig. 6a). However, induced cytokine levels were similar for cells with and without LAQ treatment (Fig. 6a). TNFα stimulation markedly increased CCL2 (*p* < 0.001) and CCL5 (*p* < 0.05) release compared to unstimulated controls. Interestingly, TNFα-stimulated cells with LAQ treatment showed higher CCL5 levels than stimulated controls (*p* < 0.01), but similar CCL2 release.

A NF-κB reporter assay was performed to assess whether LAQ modulates NF-κB activity in primary mouse microglia. After pre-treatment with 0 and 2.5 μM LAQ, microglial cells were stimulated with LPS or TNFα. Both cytokine treatments significantly increased NF-κB activity compared to unstimulated controls (*p* < 0.001 for each treatment) (Fig. 6b). The levels of NF-κB activity after LPS or TNFα stimulation did not differ between cells with (LPS: 15.2 ± 0.9, TNFα: 4.4 ± 0.6) and without LAQ treatment (LPS: 14.5 ± 1.0, TNFα: 4.6 ± 0.4) (Fig. 6b).

### LAQ decreases pro-inflammatory changes in primary astrocytes

We performed in vitro experiments in human primary astrocytes to assess whether LAQ affects astroglial morphology and secretion of pro-inflammatory factors. Pre-treatment with 250 nM LAQ inhibited reactive morphological changes characterized by reorganization of the actin cytoskeleton including a spherical cell body and highly branched processes after IL-1β- and IFNγ-stimulation (supplementary Fig. 3).

**Fig. 7:**
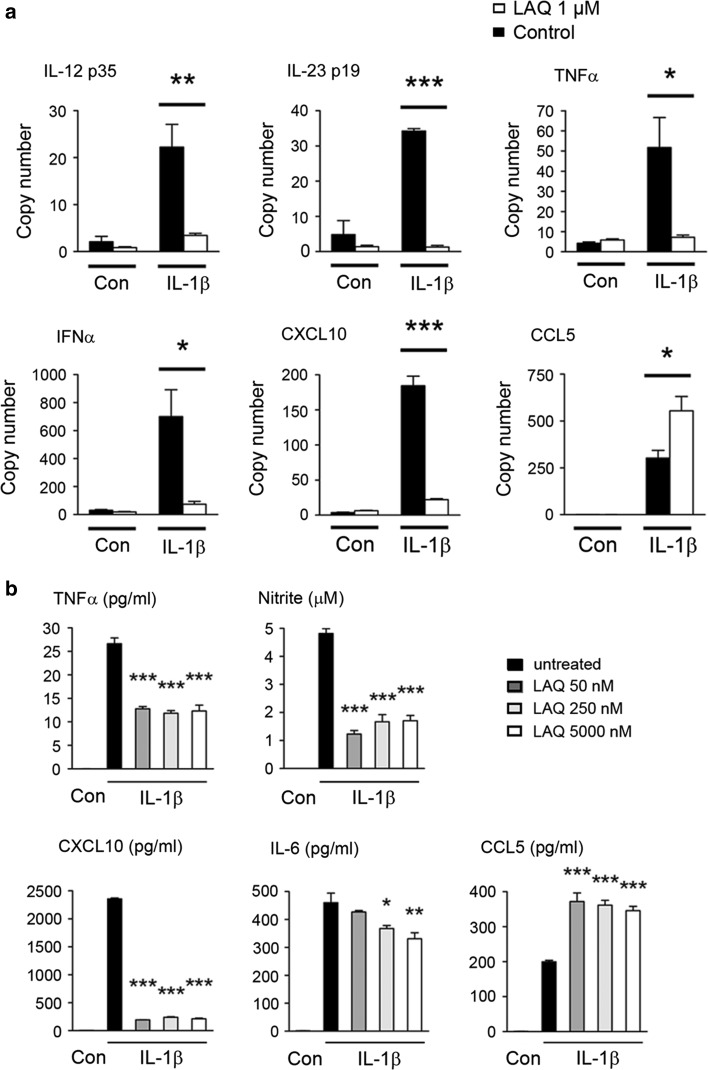
Down-modulation of inflammatory factors by LAQ treatment in vitro. LAQ reduces astrocytic inflammatory factors assessed on mRNA level by qPCR (**a**) and on protein level by ELISA and nitrite assay in supernatants (**b**). Cultures were pre-treated with LAQ and then exposed to cytokines for 24 h. For factors including TNFα, IFNα, IL-23 p19, IL-12 p35 and CXCL10, LAQ strongly reduced induction by IL-1β/IFNγ (**a**, **b**). Nitrite levels were significantly decreased by LAQ treatment (*p < 0.05, **p < 0.01, ***p < 0.001). *Graphs* show mean values with SEM from three independent experiments

To investigate the effects of LAQ on secreted pro-inflammatory factors in primary human astrocytes, subsequent qPCR and multiplex ELISA studies were performed. LAQ strongly decreased the astrocytic production of inflammatory factors on mRNA (Fig. 7a) and protein levels (Fig. 7b). LAQ strongly and significantly reduced induction in response to IL-1β (Fig. 7b) or IL-1β combined with IFNγ (Fig. 7a) for most factors examined, including TNFα, IFNα and IL-23 p19, IL-12 p35 and CXCL10. A similar effect was observed on induction of inducible nitric oxide synthase, and on resulting nitrite production (*p* < 0.001) (Fig. 7b).

### LAQ down-modulates astrocytic NF-κB activation in vivo and in vitro

The data presented here indicate that LAQ protects from cuprizone-induced demyelination through CNS-intrinsic mechanisms. Since similar protective effects have been observed in animals in which the NF-κB pathway was selectively inhibited in astrocytes [31], we further analyzed the effects of LAQ on NF-κB activation in astrocytes in vitro and in vivo.

**Fig. 8:**
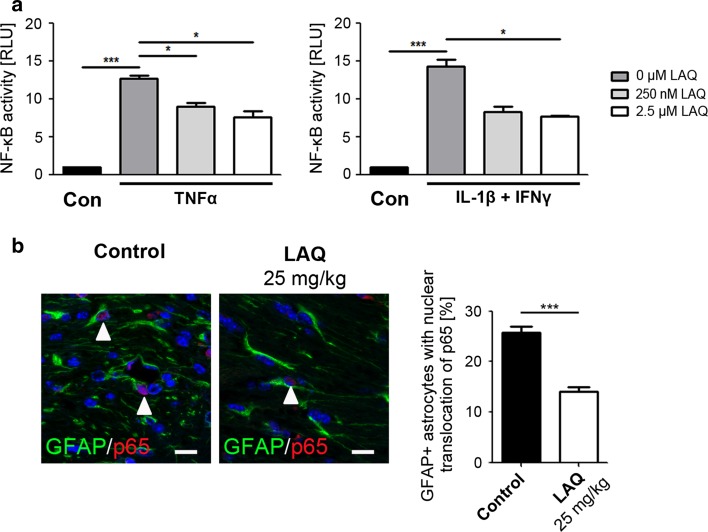
Reduction of astrocytic NF-κB activation by LAQ in vitro and in vivo. **a** Pre-treatment with LAQ reduces NF-κB activity in stimulated primary mouse astrocytes assessed by NF-κB reporter assay. Transfected cells were stimulated with IL-1β and IFNγ or TNFα alone and pre-treated with 0, 250 nM and 2.5 μM LAQ. Unstimulated cells served as controls. NF-κB activity was measured luminometrically (*RLU* relative light units). Both cytokine treatments without LAQ markedly increase NF-κB activation compared to unstimulated controls. Pre-treatment with LAQ significantly reduces NF-κB activation after cytokine stimulation. *Graphs* show mean values with SEM from three independent experiments. **b** Double immunofluorescence with antibodies to p65 (*red*) and GFAP (*green*) shows that the proportion of callosal astrocytes with nuclear p65 immunoreactivity (marked by *white arrowheads*) is significantly reduced in LAQ-treated mice (*n* = 8) (14.0 ± 0.9 %) in comparison to controls (*n* = 9) (25.8 ± 1.1 %) after 6 weeks of cuprizone (*scale bars* 10 μm) (*p < 0.05, ***p < 0.001)

To directly assess the in vitro effect of LAQ on astrocytic NF-κB activity, we examined the NF-κB activity with and without LAQ exposure in mouse astrocytes stimulated with TNFα or IL-1β combined with IFNγ. Without LAQ, both cytokine treatments significantly increased NF-κB activation compared to unstimulated controls (*p* < 0.001 for each treatment) (Fig. 8a). Pre-treatment with both LAQ doses (250 nM and 2.5 μM) significantly reduced the induced NF-κB activity after TNFα stimulation compared to stimulated controls (*p* < 0.05 for each LAQ dose; controls: 12.7 ± 0.4; 250 nM LAQ: 9.0 ± 0.5; 2.5 µM LAQ: 7.6 ± 0.8). Pre-treatment with 2.5 μM LAQ also significantly reduced NF-κB activation (7.6 ± 0.8) after stimulation with the combination of IL-1β and IFNγ compared to stimulated controls (14.2 ± 0.9, *p* < 0.05) (Fig. 8a).

To determine whether this underlying mechanism also plays a role in human astrocytes, we assessed the effect of pre-treatment with 100 nM LAQ in human astrocytes stimulated by IL-1β. In immunoblotting studies, a slowed degradation of the cytoplasmic inhibitor IκBα was observed in LAQ-treated astrocytes 5 min after IL-1β treatment (supplementary Fig. 4a). Imaging flow cytometry experiments substantiated these findings by showing that nuclear translocation of the NF-κB p65 subunit was significantly reduced by LAQ at 10 min post-IL-1β treatment (supplementary Fig. 4b).

To confirm the in vivo relevance of the above findings, we also examined astrocytic p65 translocation in the cuprizone model. Nuclear translocation of p65 was detected in astrocytes by double immunofluorescence with antibodies to p65 and GFAP. The proportion of astrocytes with nuclear p65 immunoreactivity was significantly reduced in LAQ-treated mice (Fig. 8b; 14.0 ± 0.9 %) compared to controls (25.8 ± 1.1 %, *p* < 0.001).

## Discussion

This is the first study that sheds light on the impact of LAQ on CNS-resident inflammation by investigating the effects of LAQ on cuprizone-induced demyelination in vivo and on primary CNS cells in vitro. In cuprizone-treated mice LAQ prevented demyelination, microglial activation, axonal transections, reactive gliosis and oligodendroglial apoptoses. Oligodendroglial cells were not directly affected by LAQ and microglia only displayed minor effects in vitro. In contrast, LAQ significantly reduced the inflammatory response in astrocytes by interfering with the astrocytic NF-κB activation in vivo and in vitro.

LAQ treatment significantly reduced demyelination dose-dependently after 6 weeks of cuprizone. Mice treated with 25 mg/kg LAQ showed mainly intact myelin compared to moderate demyelination with 5 mg/kg LAQ and almost complete demyelination in untreated controls. These findings were evidenced by histological and electron microscopic examination. The effect of LAQ was further associated with a 62 % reduction of apoptoses in LAQ-treated mice after 1 week of cuprizone. Importantly, acute axonal damage, the major substrate of disability in MS patients, was almost absent in LAQ-treated animals. Similar beneficial effects on axonal pathology were also observed previously in EAE [46]. Taken together, these experiments demonstrated significantly reduced tissue damage in the LAQ-treated group.

Within the CNS, LAQ-treated mice also displayed significantly less inflammation, including reduced numbers of T cells and microglia and inhibition of reactive astrogliosis. The cuprizone model is known for its minor contribution of peripheral immune system involvement in demyelination. This was further strengthened by experiments performed in Rag1^−/−^ mice, which have no T and B cells. These studies excluded any significant peripheral contribution to lesion pathology, implicating a central mechanism of action in the effects of LAQ. This is further supported by the fact that LAQ reaches the CNS even if the blood–brain barrier is intact [8]. Collectively, these findings strongly suggest that LAQ has direct protective effects on the CNS.

To date, most established therapies for MS have not shown any marked effects in the cuprizone model. Only fingolimod, a sphingosine 1-phosphate receptor modulator, led to attenuated cuprizone-induced demyelination, whereby moderate callosal demyelination was still observed in treated mice compared to complete demyelination in controls [19]. Fumaric acids did not affect demyelination or glial reactions in the cuprizone model [27]. The cuprizone model also allows study of the mechanisms of remyelination after removal of cuprizone from the diet. Fingolimod did not promote remyelination [19], whereas statins or the lack of interferon-beta even showed inhibitory effects on remyelination [26, 42]. To date, there are no reports on the effects of glatiramer acetate (GA) in this model, but GA did enhance oligodendrogenesis and remyelination in mice with lysolecithin-induced demyelination [36]. In our study, we observed nearly complete preservation of myelin and axons under preventive therapy with 25 mg/kg LAQ.

In vitro treatment of activated astrocytes with LAQ led to decreased pro-inflammatory factors. In stimulated astrocytes, LAQ reduced the levels of TNFα, IFNα, CXCL10, IL-23 p19 as well as IL-12 p35. LAQ does not have general immunosuppressive properties, but acts as an immunomodulatory substance as evidenced by the observed increased astrocytic and microglial CCL5 levels under LAQ. In vitro LAQ treatment also reduced mRNA and protein levels of CXCL10 and TNFα. Increased CXCL10 levels are mainly found in hypertrophic astrocytes surrounding inflammatory lesions in MS [39] and EAE [10]. TNFα is expressed by astrocytes, microglia and lymphocytes and is implicated in the pathogenesis of MS [34] and EAE [44]. In vivo nitrite is a final product of the reactive molecule nitric oxide. High levels of constitutive nitric oxide synthase are expressed by astrocytes in MS plaques [16]. This leads to the production of nitrite oxide and superoxide radicals which may damage oligodendrocytes, myelin sheaths, and axons [37].

Oligodendroglial viability and cell death were not directly affected by LAQ in vitro. In addition, LAQ exerted only minor effects on microglial cytokine response. Our findings regarding astrocyte-mediated changes are in accordance with a recent study demonstrating that NF-κB-dependent processes within astrocytes play a crucial role for oligodendrocyte damage during cuprizone-induced demyelination [31]. Inactivation of NF-κB in astrocytes decreased myelin loss, pro-inflammatory mediators and gliosis in the cuprizone model [31]. Reminiscent of the reduction in reactive astrogliosis seen in LAQ-treated animals, we observed prevention of characteristic morphological changes in a model of gliosis in vitro. Moreover, these therapeutically relevant concentrations of LAQ also modulated astrocytic production of factors implicated in the pathogenesis of demyelinating lesions, including TNFα and inducible nitric oxide synthase.

Astrocytic, but not microglial, NF-κB activation showed a (up to) 46 % reduction under LAQ in vitro as evidenced by NF-κB reporter assay. Inhibition of NF-κB activation by LAQ was observed rapidly within 1 h after stimulation. Similar quantitative findings on astrocytic NF-κB activation were obtained in the cuprizone model where LAQ treatment also led to a 46 % reduction of astrocytes with NF-κB activation evidenced by nuclear translocation of p65. The finding of reduced astrocytic, but similar microglial, NF-κB activation under LAQ suggests that LAQ affects upstream pathways causing NF-κB activation differentially in astrocytes and microglia.

Astroglial NF-κB activation can cause both deleterious and beneficial effects within the CNS. Protective effects include ischemia-related changes of glutamate transport [22] and increase of neurotrophic factors [49]. However, astrocytic NF-κB activation also causes deleterious effects by increasing excitotoxicity [1] and hampering neurite outgrowth [15]. Inhibition of astroglial NF-κB activation improved functional recovery after spinal cord injury [4] and EAE [5]. Taken together, these findings indicate that targeting the astrocytic NF-κB pathway might have therapeutic effects in demyelinating CNS disorders.

Modulation of the CNS-resident inflammatory response of astrocytes via NF-κB interference, as shown here for LAQ, may represent a novel protective means of restricting tissue damage and neurodegeneration in demyelinating diseases. The beneficial effects of LAQ on brain atrophy and disability progression in clinical trials may be at least partially explained by this CNS-protective effect of LAQ on neurodegeneration.

## Electronic supplementary material

Below is the link to the electronic supplementary material.

**Supplementary Fig. 1** Similar cerebral cuprizone concentration in LAQ-treated and control mice after 1 week (**a**) and 6 weeks (**b**) of 0.25% cuprizone. After 1 week (**a**) and 6 weeks (**b**) of cuprizone treatment, cuprizone was quantified in brain samples of 25 mg/kg LAQ-treated and control mice by RP-HPLC MS/MS analysis. Cuprizone concentrations are similar for both treatment regimes after both time spans (**a**, each group, n = 10, **b**, controls, n = 9; LAQ-treated mice: n = 10). Statistical analyses are performed by Mann Whitney U tests. (EPS 12241 kb)

**Supplementary Fig. 2** No weight loss in LAQ-treated Rag1^-/-^ mice. The graph shows mean body weights of control mice (n = 9) and LAQ-treated-mice (n = 10) under 0.25% cuprizone for 6 weeks. At all time points, mice treated with 25 mg/kg LAQ display higher body weights than controls. Statistical analyses are performed by Mann Whitney U tests. Data are expressed as mean values with SEM and are representative of two independent experiments. *p < 0.05. (EPS 7369 kb)

**Supplementary Fig. 3** Inhibition of morphological, reactive changes by pre-treatment with LAQ in primary astrocytes. Human astrocytes were pre-treated with or without 250 nM LAQ, then exposed to 10 ng/ml IL-1β and IFNγ for 24 h. Compared to untreated controls (left column) cytokine-treated cultures displayed cytoskeletal reorganization and morphologic changes, typical of reactive astrocytes (middle column), including a spherical cell body and multiple highly branched processes. These changes were inhibited by LAQ (right column) (*scale bars* 20 μm). Double-immunostaining for GFAP (green) and β-actin (red), counterstained with DAPI. (TIFF 25251 kb)

**Supplementary Fig. 4** Modulation of astrocyte activation by LAQ through interference with the NF-κB pathway in human primary astrocytes. (**a**) LAQ affects IL-1β activation of NF-κB activation in primary human astrocytes. Pre-treatment with 100 nM LAQ results in reduced IκBα degradation in primary human astrocyte cultures observed 5 min after IL-1β treatment. (**b**) Imaging flow cytometry experiments using human astrocyte cultures reveal that nuclear translocation of the NF-κB p65 subunit is significantly reduced by LAQ at 10 min post IL-1β treatment. Human astrocytes were exposed to 0 or 100 nM LAQ for 2 h, then washed and treated with 0 or 10 ng/ml IL-1β for10 min, fixed and stained for the NF-κB subunit p65, counterstained for Draq5 (nuclei), and subjected to imaging flow cytometry. IL-1β treatment resulted in an increase in the percentage of cells containing nuclear p65, defined as p65 and Draq co-localization. Data shown represent measurements in 20,000 cells per condition, and are typical of three studies on astrocytes from different brains. ANOVA plus Bonferroni test, **p <0.01, *p <0.05. (TIFF 6163 kb)
