# Supplementary figures and images for: Reduced astrocytic NF-κB activation by laquinimod protects from cuprizone-induced demyelination
Source: Acta Neuropathol. 2012 Jul 6;124(3):411–24. doi: 10.1007/s00401-012-1009-1 (PMC3422618; doi:10.1007/s00401-012-1009-1)

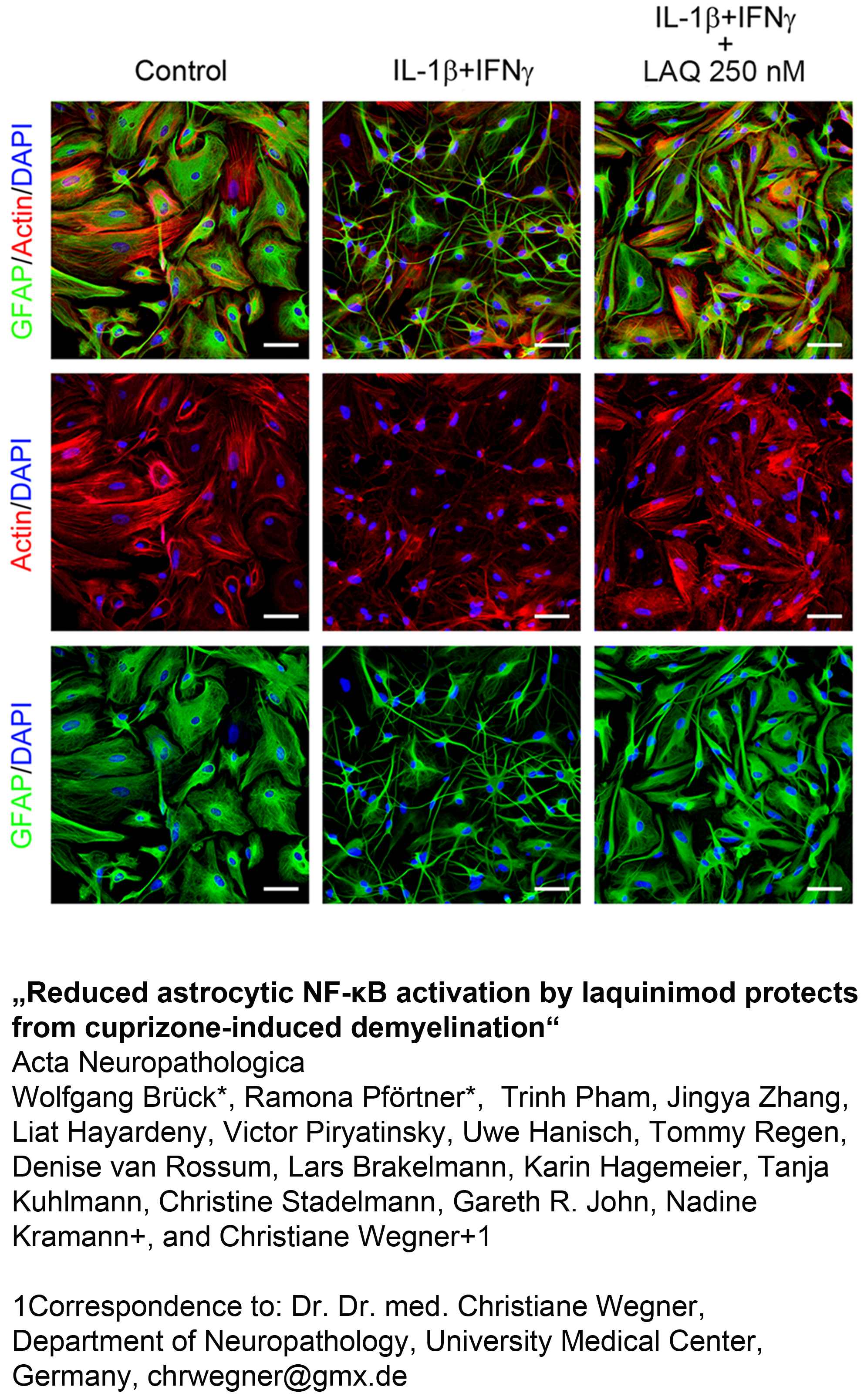

Supplement: Supplementary file 3 — Supplementary Fig. 3 Inhibition of morphological, reactive changes by pre-treatment with LAQ in primary astrocytes. Human astrocytes were pre-treated with or without 250 nM LAQ, then exposed to 10 ng/ml IL-1β and IFNγ for 24 h. Compared to untreated controls (left column) cytokine-treated cultures displayed cytoskeletal reorganization and morphologic changes, typical of reactive astrocytes (middle column), including a spherical cell body and multiple highly branched processes. These changes were inhibited by LAQ (right column) (scale bars 20 μm). Double-immunostaining for GFAP (green) and β-actin (red), counterstained with DAPI. (TIFF 25251 kb) [file 401_2012_1009_MOESM3_ESM.tif]

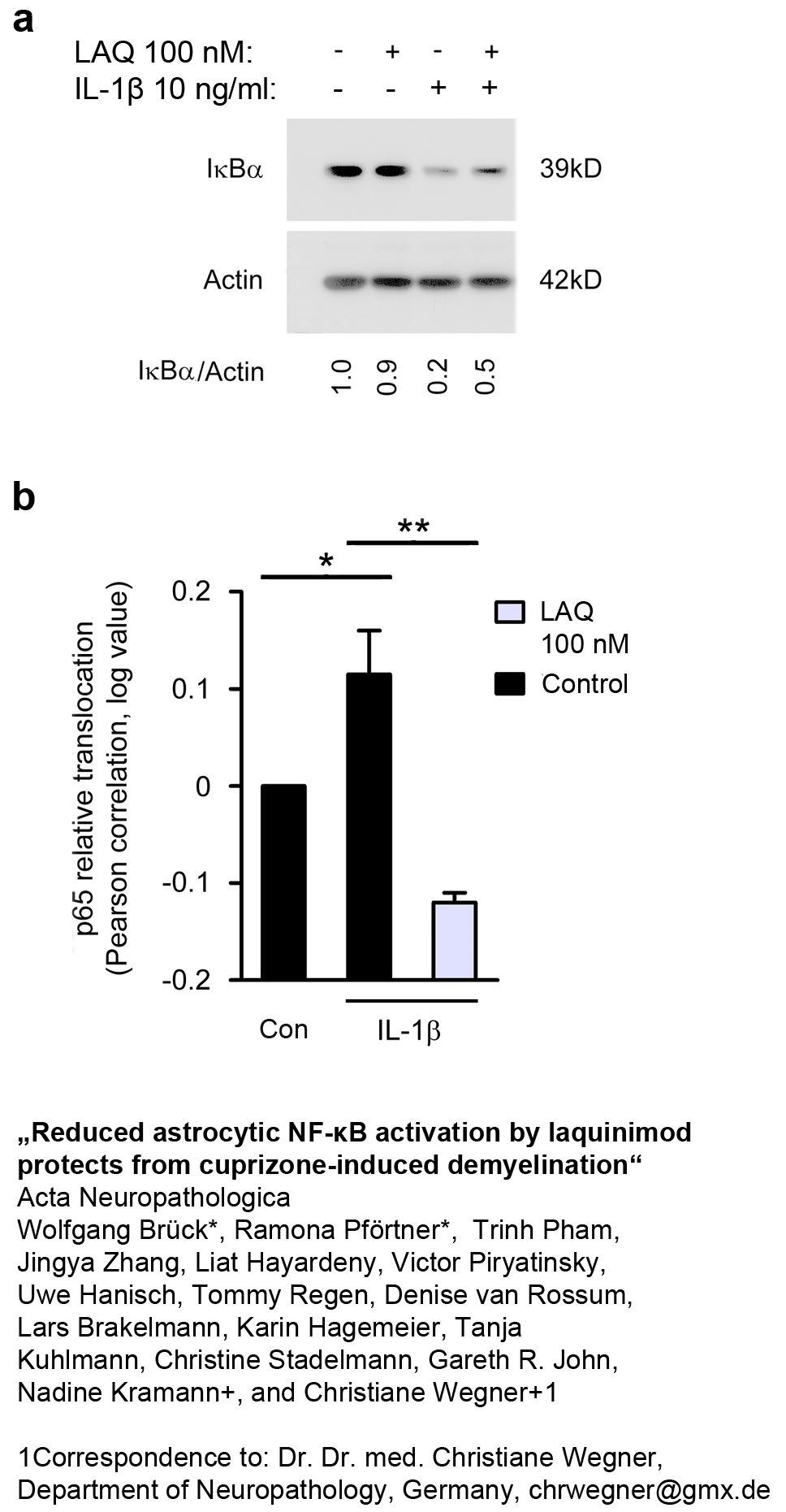

Supplement: Supplementary file 4 — Supplementary Fig. 4 Modulation of astrocyte activation by LAQ through interference with the NF-κB pathway in human primary astrocytes. (a) LAQ affects IL-1β activation of NF-κB activation in primary human astrocytes. Pre-treatment with 100 nM LAQ results in reduced IκBα degradation in primary human astrocyte cultures observed 5 min after IL-1β treatment. (b) Imaging flow cytometry experiments using human astrocyte cultures reveal that nuclear translocation of the NF-κB p65 subunit is significantly reduced by LAQ at 10 min post IL-1β treatment. Human astrocytes were exposed to 0 or 100 nM LAQ for 2 h, then washed and treated with 0 or 10 ng/ml IL-1β for10 min, fixed and stained for the NF-κB subunit p65, counterstained for Draq5 (nuclei), and subjected to imaging flow cytometry. IL-1β treatment resulted in an increase in the percentage of cells containing nuclear p65, defined as p65 and Draq co-localization. Data shown represent measurements in 20,000 cells per condition, and are typical of three studies on astrocytes from different brains. ANOVA plus Bonferroni test, **p <0.01, *p <0.05. (TIFF 6163 kb) [file 401_2012_1009_MOESM4_ESM.tif]
